# Terpenoids as principal bioactive compound of *Cissampelos oppositifolia* essential oils: enhancing synergistic efficacy with conventional antibiotics

**DOI:** 10.3389/fcimb.2024.1481656

**Published:** 2024-11-28

**Authors:** Kexin Zhao, Yurong Jiang, Kamal Dev, Xin He, Vipasha Sharma, Xinli Pang

**Affiliations:** ^1^ Department of Respiratory Medicine, Shenzhen Children’s Hospital, Shenzhen, Guangdong, China; ^2^ Shenzhen People’s Hospital (The Second Clinical Medical College, Jinan University; The First Affiliated Hospital, Southern University of Science and Technology)., Shenzhen, Guangdong, China; ^3^ Department of Pharmacology and Toxicology, Wright State University, Dayton, OH, United States; ^4^ University Institute of Biotechnology, Chandigarh University, Mohali, India

**Keywords:** terpenoids, antibiotics, synergy, spectroscopy, docking studies

## Abstract

**Background:**

The rise of antibiotic resistance imposes the search for novel antimicrobial strategies as natural products or its combination with antibiotics. This study investigates the synergistic effects of terpenoids from *Cissampelos oppositifolia* (*C. oppositifolia*) essential oil in combination with antibiotics against *Escherichia coli* (*E. coli*) and *Staphylococcus aureus* (*S. aureus*). The aims were to evaluate the antimicrobial efficacy, analyze functional group modifications and assess molecular interaction.

**Methods:**

Essential oil was extracted from *C. oppositifolia* by hydro-distillation. The EO was analyzed for terpenoid content via Thin Layer Chromatography (TLC). Antimicrobial activity was assessed using the disc diffusion method and Minimum Inhibitory Concentration determinations (MIC) by broth dilution followed by bactericidal essay (Time-killing). FTIR and UV spectroscopy were employed to detect functional group modifications in terpenoid-antibiotic combinations. Molecular docking studies assessed interaction energies between terpenoids and antibiotics.

**Results:**

TLC identified α-pinene, δ-carene, and caryophyllene in the EO. δ-Carene exhibited the highest synergy with antibiotics, showing the lowest MIC of 0.04 mg/mL against *S. aureus* ATCC-43300 and 0.05 mg/mL against *E. coli* MTCC-739. Time-kill assays demonstrated that α-pinene, δ-carene, and caryophyllene achieved complete bacterial eradication by 4 hours in combination with amoxicillin against *E. coli*, and by 2 hours against *S. aureus* in combination with erythromycin. FTIR analysis revealed peak shifts at 1599, 1774, and 2259 cm^−1^ for amoxicillin + α-pinene, and new peaks at 1648 and 1287 cm^−1^ for δ-carene + erythromycin. UV spectra indicated potential complex formations. Docking studies showed δ-carene’s strong interaction with erythromycin and amoxicillin, with interaction energies of -96.10 and -87.75 kcal/mol, respectively.

**Conclusion:**

Terpenoids from *C. oppositifolia* enhance the antimicrobial efficacy of antibiotics. Functional group modifications and complex formations suggest that these interactions may contribute to synergistic effects. These findings support the potential use of terpenoid-antibiotic combinations in overcoming antibiotic resistance and warrant further investigation into their mechanisms of action.

## 1 Introduction

The global incidence of microbial infectious diseases is rising (Masyita et al., 2022; Salvi et al., 2022; Baker et al., 2022), significantly impacted by the increasing prevalence of microbial drug resistance to current antimicrobial agents (Salam et al., 2023). This resistance results in multidrug-resistant microbes causing widespread infections. This is linked to higher morbidity and mortality rates of about 4.95 million people who died by drug-resistant infections among 1.27 million deaths by antibiotic resistance in 2019 (Antimicrobial Resistance Collaborators, 2022). The growing issue of antibiotic resistance and the higher recurrence rates of common infections are straining healthcare systems and societies (Ali et al., 2024). Predictions indicate that by 2050, the mortality attributable to antimicrobial resistance may exceed 10 million annually, surpassing that of other major diseases and cancers (de Kraker et al., 2016). Consequently, there is an urgent need for novel antimicrobial agents or the use of a combination of natural remedies with modern antibiotics to treat MDR infections (Konwar et al., 2022). Natural products, particularly those derived from plants, offer a promising avenue for discovering new antimicrobial agents (Cowan, 1999). Medicinal plants have been used throughout history for their therapeutic properties, with essential oils (EOs) being one of the most prominent natural products and so catching the attention of researchers globally (Nasim et al., 2022; Chaachouay and Zidane, 2024). EOs are concentrated hydrophobic liquids derived from plants, known as rich sources of bioactive compounds with antimicrobial, antiviral, antioxidant, anti-inflammatory, and other pharmacological effects. Plants produce these chemicals to protect themselves, contributing to their color, aroma, and flavor. Essential oils, as key secondary metabolites, are responsible for many of these pharmaceutical properties (Masyita et al., 2022; Salvi et al., 2022). EOs are found in the cytoplasm of specific plant cell secretions in various plant organs, including secretory hairs or trichomes, epidermal cells, internal secretory cells, and secretory pockets (Gersbach, 2002). These oils are complex mixtures that may contain over 300 different compounds. They consist of organic volatile compounds, generally of low molecular weight below 300 g/mol (Dhifi et al., 2016; de Sousa et al., 2023). These Eos consistent compounds belong to various chemical classes: alcohols, ethers or oxides, aldehydes, ketones, esters, amines, amides, phenols, heterocycles, and mainly the terpenes. Alcohols, aldehydes, and ketones offer a wide variety of aromatic notes, such as fruity ((E)-nerolidol), floral (Linalool), citrus (Limonene), herbal (γ-selinene), etc. Among all chemical components of EOs, terpenes and terpenoids have been comprehensively studied and reported to play key roles in human health (Jugreet et al., 2020; Haro-González et al., 2021). Terpenoids or terpenes are the most structurally varied classes and they are the largest family of compounds of plant products. Previous studies estimated the existence of more than 23,000 known terpenoid compounds, including monoterpenes, sesquiterpene, carotenoids, tetraterpenes, diterpenes, tocopherol, phytol, steroids and hormones (Masyita et al., 2022). The monoterpenes and sesquiterpene are the most dominant terpenoids present in the Eos. These compounds contribute to the flavor and aroma of plants but they have therapeutic potential (Proshkina et al., 2020). The monoterpenes and sesquiterpene oxygenated derivatives of terpenes are recognized for their ability to combat both antibiotic-susceptible and resistant bacteria. They act primarily by disrupting microbial cell membranes, inhibiting protein synthesis, and interfering with DNA replication (Masyita et al., 2022). Compounds such as carvacrol, carvone, eugenol, geraniol, and thymol have demonstrated antibacterial properties against pathogens including *Staphylococcus aureus*. *C. oppositifolia* is a traditional medicinal plant widely used in various cultures for its therapeutic properties. Known for its rich phytochemical profile, it contains essential oils abundant in terpenoids, particularly α-pinene, δ-carene, and caryophyllene, which are recognized for their antimicrobial, anti-inflammatory, and antioxidant activities (Shang et al., 2024). Historically, the plant has been employed in herbal medicine to treat ailments such as fevers, respiratory disorders, and infections (Sharma et al., 2021). Recent studies highlight its potential in modern medicine, especially in enhancing the efficacy of conventional antibiotics, making it a promising candidate for further research in phytotherapy and drug development (Mahizan et al., 2019). These terpenoids have shown potential in enhancing the effectiveness of conventional antibiotics through synergistic interactions. This study aims to investigate the principal bioactive terpenoids in the essential oils of *C. oppositifolia* and their role in improving antibiotic efficacy. By exploring these interactions, the research seeks to contribute to the development of more effective antimicrobial strategies and address the growing challenge of drug-resistant infections.

## 2 Methods

The present study was conducted in accordance with the biosafety regulations of the university. *Escherichia coli* MTCC-739 and *Staphylococcus aureus* ATCC-43300 were obtained from the Institute of Microbial Technology (IMTECH), Chandigarh. The research was approved by the university’s research committee.

### 2.1 Plant material and antimicrobial property screening

A total of 72 plant specimens (**Supplementary Table 1**), including *C. oppositifolia*, were collected from two locations in Himachal Pradesh, India: Solan (altitude 1350 m, temperature 20-30°C, humidity 55-68%) and Shimla (altitude 2202 m, temperature 12-25°C, humidity 62-80%). The specimens, comprising leaves, buds, flowers, and tubers, were identified and verified using the herbarium at Dr Y.S. Parmar University of Horticulture and Forestry, Nauni, Himachal Pradesh. The collected specimens were washed with water and surface-sterilized using 1% hydrogen peroxide (H_2_O_2_), followed by rinsing with autoclaved distilled water to remove any residual H_2_O_2_, as per an established protocol in our laboratory. The surface-sterilised plant material was then dried in an oven at 40°C until completely desiccated. The dried material was ground into a fine powder using an electronic mixer grinder (Sharma and Mishra, 2019). Among the specimens, *C. oppositifolia* extract exhibited significantly higher antimicrobial activity, as shown in our published data. Phytochemical analysis of the extracts revealed the presence of various bioactive compounds, including phenols, terpenoids, flavonoids, tannins, coumarins, glycosides, and alkaloids. EOs were subsequently extracted from *C. oppositifolia* and further investigated for this study.

### 2.2 Extraction of essential oil

The EOs were extracted using an environmentally friendly hydro-distillation method (Abbas et al., 2022) and calculated by Extract Yield (%)= (Weight of starting material/Weight of extract obtained)×100. Fresh leaves and inflorescence of *C. oppositifolia* were subjected to hydro-distillation using a Clevenger apparatus. Two hundred grams of chopped plant material were placed in a round-bottom flask, and 400 mL of distilled water was added. The mixture underwent hydro-distillation for 3 hours. Afterwards, the organic phase was dried over sodium sulphate, and filtered, and the solvent was evaporated to dryness. The volatile oil was then stored at 4°C until analysis. This method combines extraction and steam distillation into a single process, which reduces trial time, saves solvents, simplifies equipment, and facilitates operation. While the extraction rate is relatively low, the essential oil obtained is of high purity. However, the process involves high temperatures, which may lead to the volatilization or oxidation of certain components in the EOs. The method primarily collects insoluble EOs, resulting in the loss of water-soluble components and, consequently, a lower extraction yield. The extracted oil is still a multi-component composite essential oil, making it challenging to achieve selective extraction and complete purity.

### 2.3 Extraction of terpenoids

The EOs were further processed to isolate terpenoids using TLC (Jiang et al., 2016). Initially, a small quantity of the essential oil was dissolved in a minimal amount of an extraction solvent, typically a mixture of hexane and ethyl acetate in a 7:3 ratio. This solution was then placed onto a pre-coated silica gel TLC plate, which had been activated by heating at 110°C for 30 minutes. The TLC plate was placed in a development chamber containing a mobile phase of hexane and ethyl acetate (7:3 ratio), allowing the solvent front to rise to about 3/4th of the plate’s height. After development, the TLC plate was removed from the chamber and air-dried. Visualization of the terpenoids was achieved by observing the plate under UV light at 254 nm and 365 nm, where terpenoids typically have fluorescence. For further visualization, the plate was sprayed with anisaldehyde-sulfuric acid reagent and heated at 110°C for 10 minutes, revealing distinct colored bands corresponding to the terpenoids. The retention factor (Rf) values of these bands were calculated and compared with known standards to identify the specific terpenoids present in the essential oil α-pinene, δ-carene, and caryophyllene. The results were documented by photographing the TLC plates and noting the colors and Rf values of the terpenoid spots.

### 2.4 Antimicrobial and synergistic effect of α-pinene, δ-carene, caryophyllene

#### 2.4.1 Antimicrobial susceptibility testing

The antimicrobial susceptibility of the terpenoids—α-pinene, δ-carene, and caryophyllene, was assessed using the disc diffusion method (Gupta et al., 2023). Each compound was dissolved in dimethyl sulfoxide (DMSO) at a concentration of 0.75 mg/mL and stored in sterile centrifuge tubes at 4°C to reduce volatilization. For the susceptibility tests, the stock solutions were further diluted to 0.25 mg/mL, with 0.25% DMSO serving as the control. Bacterial inoculation was standardized to a concentration of 0.5 McFarland (1.5×10^5^ CFU/mL) by spectrophotometric measurement at 625 nm, ensuring an optical density of 0.06. The terpenoids were tested against *E. coli* MTCC-739 and *S. aureus* ATCC-43300. Standard antibiotics, amoxicillin (30 µg) and erythromycin (15 µg) were included for comparison, both individually and in combination with each terpenoid. Mueller-Hinton agar plates were inoculated and incubated at 37°C for 18-20 hours. Antimicrobial activity was evaluated by measuring the zone of inhibition around each disc. Compounds that exhibited significant antimicrobial activity were selected for further determination of MIC.

#### 2.4.2 Minimum inhibitory concentration determination

MIC values were determined using the microdilution method (Kowalska-Krochmal and Dudek-Wicher, 2021), with concentrations of each terpenoid ranging from 0.25 to 0.002 mg/mL. Assays were conducted individually for each microorganism to prevent cross-interaction due to volatilization. All tests were performed in triplicate, and results were interpreted according to the CLSI-M100 guidelines (Clinical & Laboratory Standards Institute, 1999).

#### 2.4.3 Time-kill study

A 24-hour time-kill curve study was performed according to CLSI document M26-A guidelines (M26-A Methods for Determining Bactericidal Activi.pdf, 2024). The terpenoids were evaluated at MIC, 2xMIC, and 4xMIC concentrations, with a negative control consisting of Mueller-Hinton broth, 0.25% DMSO, and bacterial inoculum. Bacterial suspensions were prepared as previously described, and the samples were incubated at 35°C for 24 hours. At intervals of 0, 2, 4, 8, 12, and 24 hours, 10 µL of each sample was diluted in 990 µL of 0.9% sterile saline, and 100 µL of this dilution was plated onto Mueller-Hinton agar. Plates were incubated for 24 hours at 35°C, after which colony-forming units (CFU) were manually counted. The counts were multiplied by 1000 to determine CFU/mL, and these values were transformed into a logarithmic scale to generate time-kill curves. A bactericidal effect was defined as a ≥ 3 log_10_ reduction in CFU from the initial count.

### 2.5 FTIR spectra of terpenoids and combination with antibiotics

FTIR spectroscopy was employed to investigate the molecular interactions between terpenoids (α-pinene, δ-carene, and caryophyllene) and antibiotics-amoxicillin and erythromycin. Terpenoids and antibiotics were prepared in solution, and FTIR spectra were recorded over the range of 4000–400 cm^−1^ using an ATR accessory for liquid samples and KBr pellets for solid samples. The spectra were analyzed for characteristic peaks and shifts to identify functional groups and potential interactions. Comparison of the spectra of individual terpenoids with their combinations with antibiotics revealed changes in peak positions or intensities, providing insights into molecular interactions and potential synergistic effects.

### 2.6 UV spectrophotometer spectra of terpenoids and combinations with antibiotics

UV spectrophotometry was utilized to analyze the absorbance characteristics of terpenoids-α-pinene, δ-carene, and caryophyllene and their combinations with antibiotics-amoxicillin and erythromycin. Solutions of each terpenoid and antibiotic were prepared at a concentration of 0.1 mg/mL in methanol or acetonitrile. Combined samples were also prepared by mixing equal volumes of the terpenoid and antibiotic solutions. Using a UV-visible spectrophotometer, spectra were recorded over the range of 200–400 nm. The baseline spectrum of the solvent was first recorded to correct for any background absorbance. Each sample was then analyzed to identify characteristic absorbance peaks and any shifts or changes when terpenoids were combined with antibiotics. These changes provided insights into the interactions between the compounds and their potential synergistic effects.

### 2.8 Docking study of combinations of terpenoids with antibiotics

The chemical structures of the selected terpenoids α-pinene, δ-carene, and caryophyllene and antibiotics-erythromycin and amoxicillin were first retrieved from the PubChem database- https://pubchem.ncbi.nlm.nih.gov/in.sdf format and converted to.pdb format using OpenBabel. These ligands were then subjected to energy minimization using the MMFF94 force field to obtain their most stable conformations, with protonation states adjusted and any missing hydrogens added.

## 3 Results

Of 71 plant *C. oppositifolia* extracts that showed interesting antimicrobial potential, further essential oil was extracted. The EO by hydro-distillation method yielded 1.2% by weight of the starting plant material. The purity of the extracted oil was measured at 95%, but the process led to the loss of approximately 10% of volatile components and the oxidation of some sensitive compounds. The oil primarily contained insoluble components, with a reduction of 15% in water-soluble substances. Despite these losses, the EO maintained a high degree of purity and was suitable for further analysis.

### 3.1 Terpenoids and antibiotics

**Figure 1:**
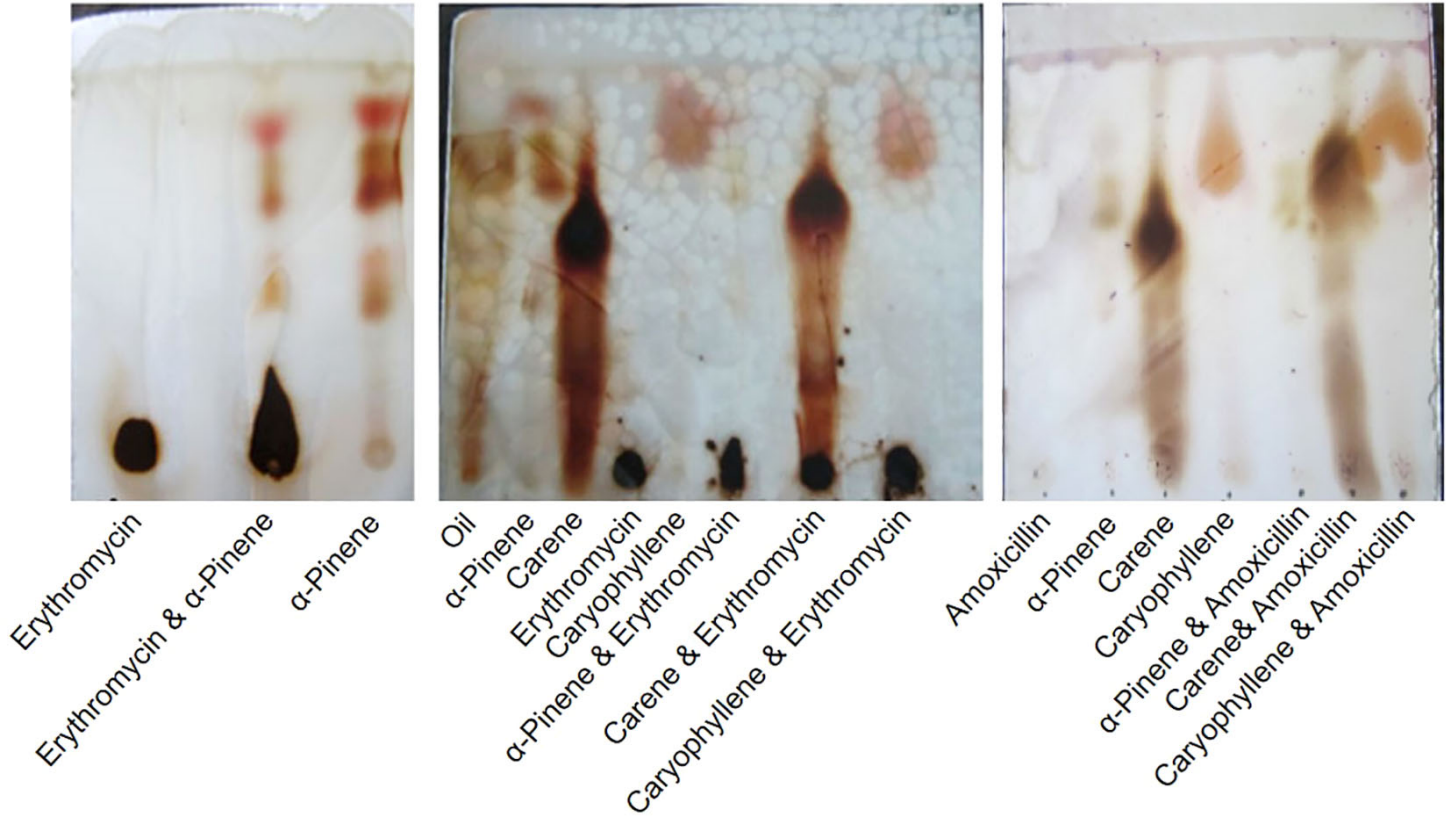
Thin Layer Chromatography (TLC) Analysis of Terpenoids from *Cissampelos oppositifolia* Extracts and Their Combination with Antibiotics.

The TLC analysis of the EO from *C. oppositifolia* revealed the presence of several terpenoids, including monoterpenes- α-pinene, δ-carene, and sesquiterpene-caryophyllene (**Figure 1**). These terpenoids were identified based on their characteristic retention factor (Rf) values compared to known standards. The terpenoids were tested in combination with two antibiotics, amoxicillin and erythromycin (**Figure 1**).

### 3.2 Antimicrobial susceptibility

The antimicrobial screening results for *S. aureus* ATCC-43300 and *E. coli* MTCC-739 demonstrated varying degrees of efficacy for the essential oil terpenoids and their combinations with antibiotics (**Table 1**). For *S. aureus* ATCC-43300, erythromycin alone yielded a zone of inhibition of 7 ± 0.2 mm. When tested individually, α-pinene also showed a zone of 7 ± 0.2 mm, while δ-carene and caryophyllene produced zones of 10 ± 0.2 mm and 8 ± 0.2 mm, respectively. The combination of terpenoids with erythromycin enhanced the inhibitory effect, with α-pinene + erythromycin producing a zone of 10 ± 0.2 mm, δ-carene + erythromycin showing the largest zone of 11 ± 0.2 mm, and caryophyllene + erythromycin yielding 8 ± 0.2 mm. Among these, δ-carene exhibited the most significant synergistic effect with erythromycin against *S. aureus*. For E. coli MTCC-739, amoxicillin alone resulted in a zone of inhibition of 4 ± 0.2 mm. The individual terpenoids showed improved activity, with α-pinene, δ-carene, and caryophyllene producing zones of 9 ± 0.2 mm, 11 ± 0.2 mm, and 7 ± 0.2 mm, respectively. Combinations of terpenoids with amoxicillin led to further enhanced inhibition: α-pinene + amoxicillin resulted in a zone of 12 ± 0.2 mm, δ-carene + amoxicillin showed a zone of 11 ± 0.2 mm, and caryophyllene + amoxicillin achieved 8 ± 0.2 mm. The combination of α-pinene with amoxicillin exhibited the most significant synergistic effect against *E. coli*.

**Table 1:** Antimicrobial activity of terpenoids alone and in combination with antibiotics against *Staphylococcus aureus* ATCC-43300 and *Escherichia coli* MTCC-739.

Antibiotic/Terpenoid	*Staphylococcus aureus* ATCC-43300	*Escherichia coli* MTCC-739
	Zone of Inhibition (mm)	Zone of Inhibition (mm)
Erythromycin	7 ± 0.2	–
α-Pinene	7 ± 0.2	9 ± 0.2
Carene	10 ± 0.2	11 ± 0.2
Caryophyllene	8 ± 0.2	7 ± 0.2
α-Pinene + Erythromycin	10 ± 0.2	–
δ-carene + Erythromycin	11 ± 0.2	–
Caryophyllene + Erythromycin	8 ± 0.2	–
Amoxicillin	–	10± 0.2
α-Pinene + Amoxicillin	–	12 ± 0.2
δ-carene + Amoxicillin	–	11 ± 0.2
Caryophyllene + Amoxicillin	–	8 ± 0.2
Best Synergistic Effect with Erythromycin	δ-carene + Erythromycin	–
Best Synergistic Effect with Amoxicillin	–	- α-Pinene + Amoxicillin

#### 3.2.1 MICs of terpenoids

MICs of the compounds were evaluated for both *S. aureus* ATCC-43300 and *E. coli* MTCC-739. The results are presented in **Table 2**. α-Pinene exhibited a MIC of 0.08 mg/mL against S. aureus ATCC-43300 and 0.06 mg/mL against E. coli MTCC-739, indicating a moderate inhibitory effect. δ-carene showed the lowest MIC values, with 0.04 mg/mL for *S. aureus* ATCC-43300 and 0.05 mg/mL for *E. coli* MTCC-739, suggesting a stronger antimicrobial activity compared to the other compounds. Caryophyllene had MICs of 0.07 mg/mL against *S. aureus* ATCC-43300 and 0.08 mg/mL against *E. coli* MTCC-739, reflecting its effective but slightly less potent activity. Erythromycin had an MIC of 0.5 mg/mL against *S. aureus* ATCC-43300, while amoxicillin showed an MIC of 0.5 mg/mL against *E. coli* MTCC-739. These results highlight the varying levels of effectiveness of the terpenoids and antibiotics against the tested bacterial strains.

**Table 2:** Minimum Inhibitory Concentrations (mg/mL) of the terpenoids.

Compound	*S. aureus* ATCC-43300	*E. coli* MTCC-739
α-Pinene	0.08	0.06
Carene	0.04	0.05
Caryophyllene	0.07	0.08
Erythromycin	0.5	N/A
Amoxicillin	N/A	0.5

#### 3.2.2 Time killing

**Figure 2:**
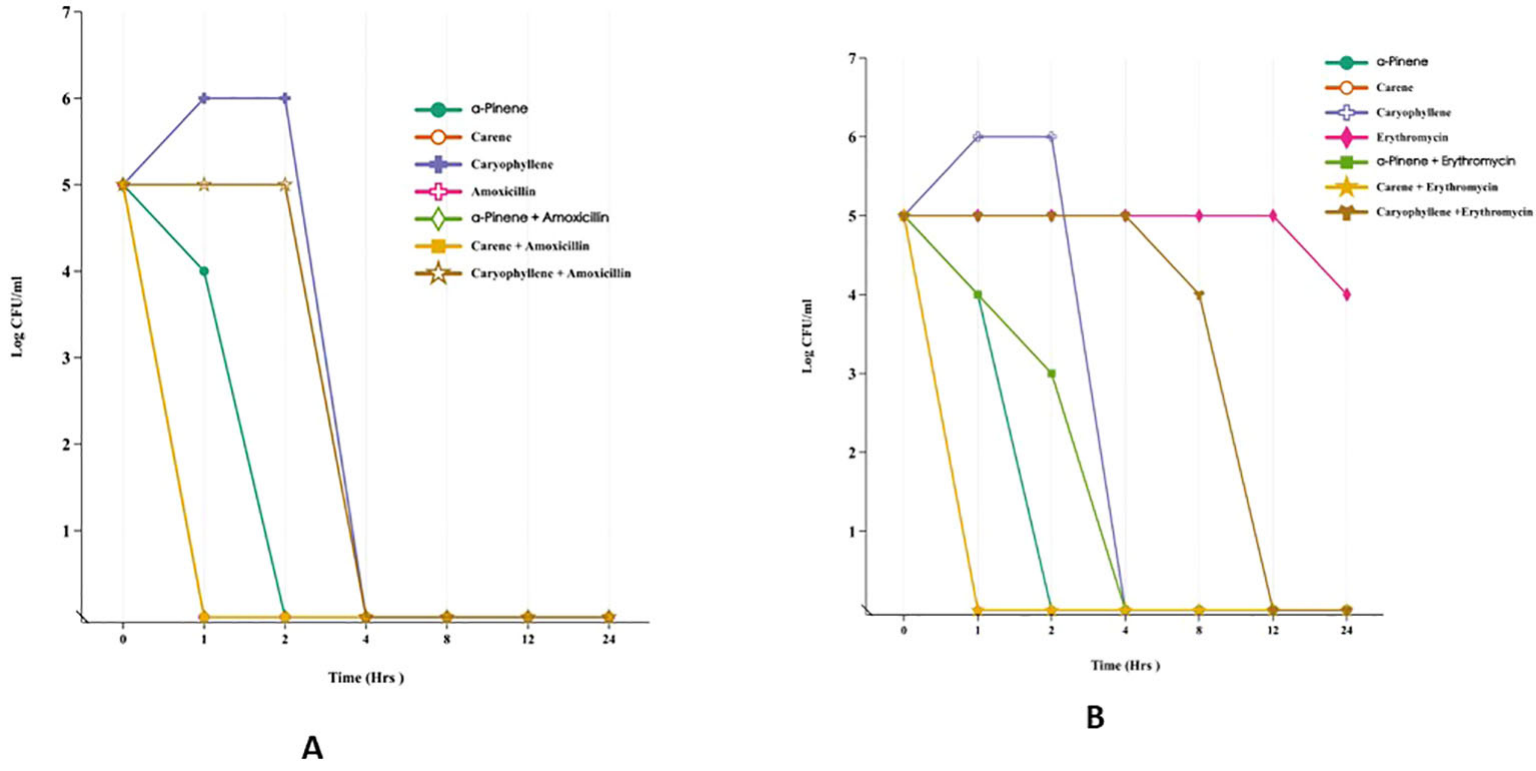
Time-Kill Assay of Terpenoids and Their Combinations with Antibiotics Against *E*. *coli* MTCC-739 **(A)** and *S. aureus* ATCC-43300 **(B)**.

he time-kill assay was conducted to assess the antimicrobial efficacy of α-pinene, δ-carene, and caryophyllene against *E. coli* MTCC-739 and *S. aureus* ATCC-43300, both individually and in combination with amoxicillin or erythromycin (**Figures 2A, B**). All treatments began with an initial bacterial load of 5 log_10_ CFU/ml at time zero. In the case of *E. coli*, during the first hour, α-pinene, δ-carene, and caryophyllene alone showed varying antibacterial activity, with bacterial counts reducing to 4, 0, and 6 log_10_ CFU/ml, respectively. Amoxicillin alone did not reduce the bacterial load at this early stage. By the second hour, α-Pinene and δ-carene maintained complete bacterial killing (0 log_10_ CFU/ml), while caryophyllene remained less effective, with a count of 6 log_10_ CFU/ml. Amoxicillin alone continued to show no reduction. By the four-hour mark, all treatments, including the combinations of α-pinene + amoxicillin, δ-carene + amoxicillin, and caryophyllene + amoxicillin, achieved a complete bacterial kill, maintaining a 0 log_10_ CFU/ml count. This bactericidal effect persisted through 8, 12, and 24 hours, indicating the potent efficacy of these compounds, particularly when combined with amoxicillin. A similar pattern was observed in *S. aureus* ATCC-43300. After 1 hour, α-Pinene and δ-carene alone reduced the bacterial load to 4 log_10_ CFU/ml, while caryophyllene alone led to an increase in the bacterial count to 6 log_10_ CFU/ml. Erythromycin alone showed no significant reduction, but the combinations of α-pinene + erythromycin and δ-carene + erythromycin completely eradicated the bacteria, achieving 0 log_10_ CFU/ml. By the second hour, α-Pinene alone eliminated *S. aureus*, while δ-carene reduced the count to 2 log_10_ CFU/ml. Caryophyllene remained ineffective with 6 log_10_ CFU/ml, and erythromycin alone was still ineffective. At the four-hour mark, all treatments, except caryophyllene and erythromycin alone, achieved complete bacterial killing. This trend continued through 8, 12, and 24 hours, with α-pinene, δ-carene, and their combinations with erythromycin effectively maintaining a 0 log_10_ CFU/ml count. In contrast, caryophyllene and erythromycin alone exhibited limited antibacterial activity, with caryophyllene showing no reduction and erythromycin gradually reducing the bacterial load to 4 log_10_ CFU/ml by 24 hours. These results demonstrate that α-pinene, δ-carene, and their combinations with either amoxicillin or erythromycin exhibit strong bactericidal activity against both *E. coli a*nd *S. aureus*. To further validate these primary results, it is essential to investigate the mode of action of each terpenoid—α-pinene, δ-carene, and caryophyllene—by studying their effects on bacterial cell wall integrity. This can be done through assays designed to assess cell wall disruption, which would provide insights into the bactericidal mechanisms of these compounds. By evaluating how these terpenoids interact with bacterial cell walls, we can better understand whether their antimicrobial efficacy stems from cell wall destruction.

### 3.3 FTIR spectroscopy

**Figure 3:**
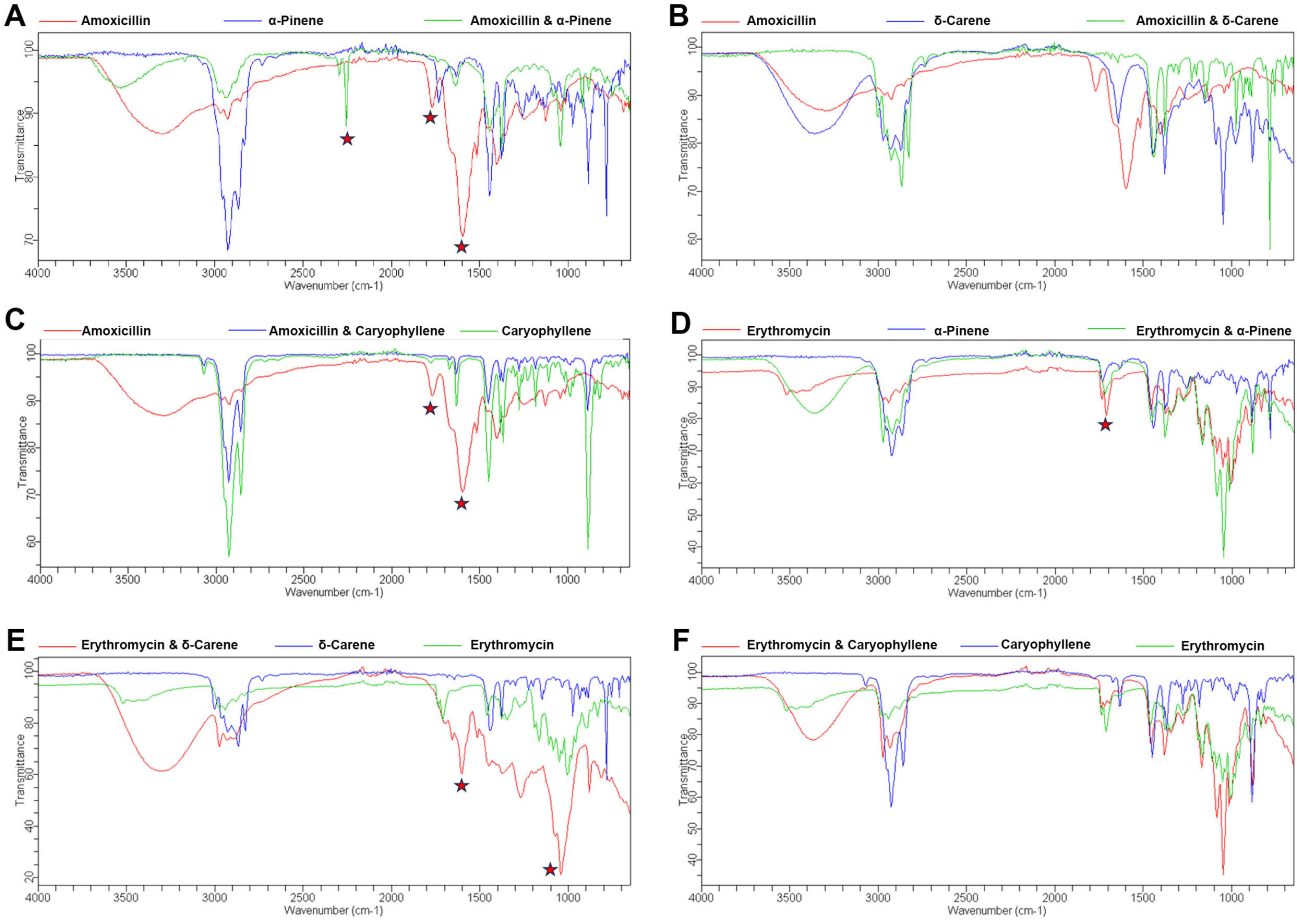
FTIR Spectroscopy Analysis of Functional Group Modifications in Terpenoid-Antibiotic Combinations. **(A)** Amoxicillin + α-Pinene; **(B)** Erythromycin + α-Pinene; **(C)** Amoxicillin + δ-Carene **(D)** Amoxicillin + Caryophyllene **(E)** Erythromycin + δ-Carene **(F)** Erythromycin + Caryophyllene.

The FTIR spectra analysis of antibiotics and terpenoids (**Supplementary Figure 1**) revealed significant modifications in the functional groups when combined, suggesting potential synergistic interactions. The overlay spectra for the combinations were analyzed. For the amoxicillin + α-Pinene combination, distinct peak shifts were observed at 1599, 1774, and 2259 cm^−1^ (**Figure 3A**). Similarly, the combination of α-Pinene with Amoxicillin showed a peak shift at 1715 cm^−1^ (**Figure 3B**). The combination of amoxicillin with caryophyllene exhibited peak shifts at 1599 and 1774 cm^−1^ (**Figure 3C**). Notably, the overlay of caryophyllene with amoxicillin resulted in similar peak modifications as seen with carene, at 1599 and 1774 cm^−1^ (**Figure 3D**). In contrast, the combination of δ-carene with erythromycin generated new peaks at 1648 and 1287 cm^−1^, indicating a different interaction pattern (**Figure 3E**). Furthermore, the overlay of caryophyllene with amoxicillin led to the disappearance of the amoxicillin peaks at 1599 and 1774 cm^−1^, while no modifications were observed in the caryophyllene and erythromycin combination (**Figure 3F**). These spectral changes suggest that the combination of terpenoids with antibiotics results in functional group modifications, which could be responsible for the observed synergistic effects in antimicrobial activity.

### 3.4 UV spectrophotometer

**Figure 4:**
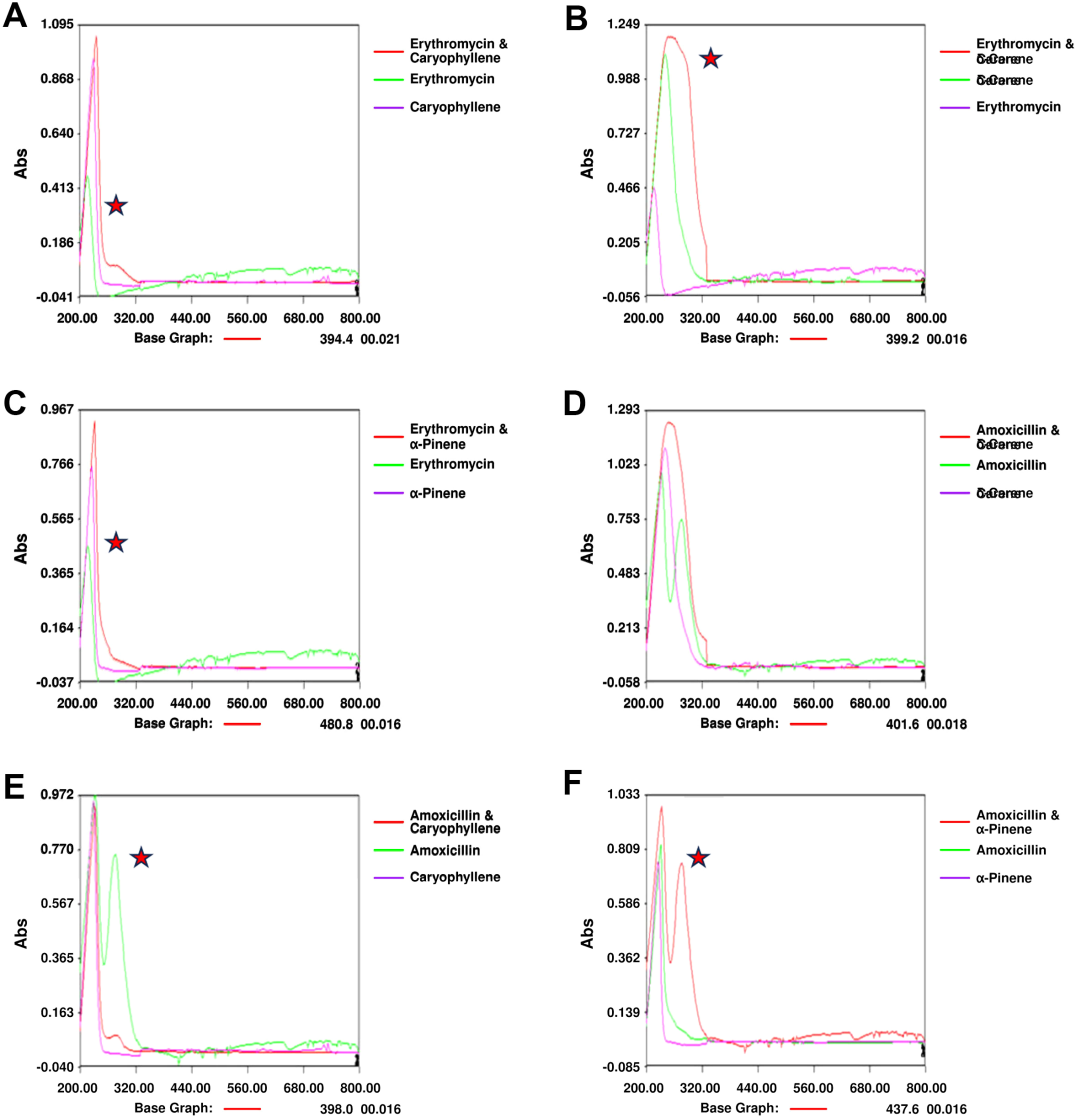
UV Spectrophotometry Analysis of Terpenoid-Antibiotic Interactions. **(A)** Amoxicillin + α-Pinene; **(B)** Erythromycin + α-Pinene; **(C)** UV Spectrum of Amoxicillin + δ-Carene; **(D)** Amoxicillin + Caryophyllene; **(E)** UV Spectrum of Erythromycin + δ-Carene; **(F)** Erythromycin + Caryophyllene.

Notable changes in the UV spectra were observed when terpenoids were mixed with antibiotics, suggesting possible interactions or alterations in the molecular structure of the components. In the combination of amoxicillin with α-Pinene (**Figure 4A**), significant shifts in the absorption peaks were noted, indicating potential interactions that may alter the electronic environment of the molecules. These shifts could be indicative of changes in the conjugation of the antibiotic’s chromophore when combined with the terpenoid. Similarly, the combination of erythromycin with α-Pinene (**Figure 4B**) also showed noticeable peak shifts, further supporting the possibility of complex formation between these molecules. When amoxicillin was combined with δ-carene (**Figure 4C**), distinct modifications in the absorption spectra were observed, highlighting potential changes in the molecular interactions. Caryophyllene, when mixed with amoxicillin (**Figure 4D**), showed similar alterations in the UV spectra, suggesting that the interaction between these molecules may involve modifications in their electronic structures. The combination of δ-carene and erythromycin (**Figure 4E**) revealed the formation of new absorption peaks, indicating the possible creation of new molecular entities or complexes. Finally, the spectra for caryophyllene and erythromycin (**Figure 4F**) showed minimal modifications, suggesting that while interactions may occur, they might be less pronounced than with other combinations. Overall, these results suggest that the mixing of terpenoids and antibiotics leads to changes in their UV absorption spectra, which could indicate complex formation or alterations in their molecular structures. These findings provide valuable insights into the potential synergistic effects observed in combination therapies involving these compounds.

### 3.5 Docking interactions of terpenoids with antibiotics

**Figure 5:**
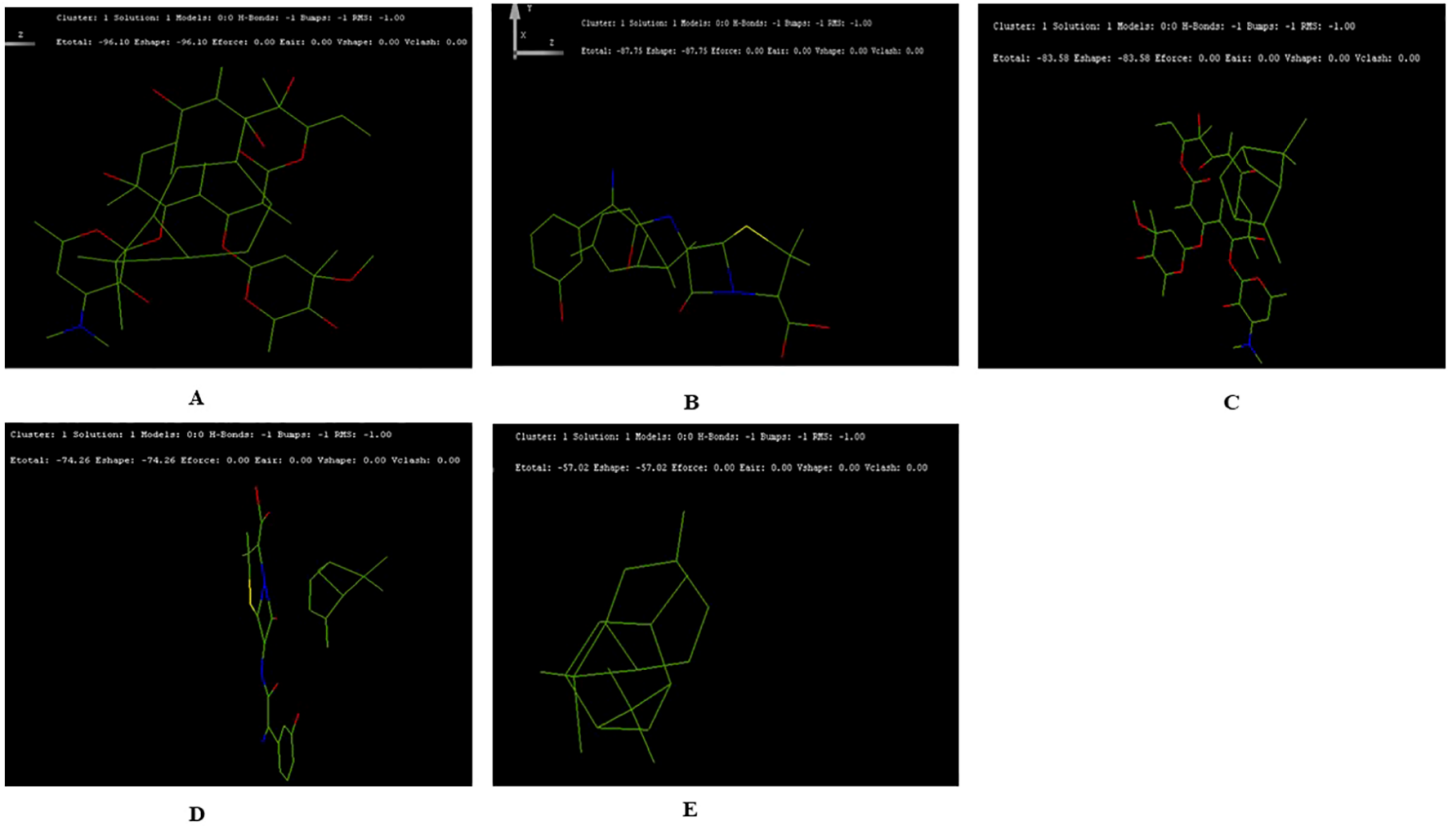
Docking Interactions of Terpenoids with Antibiotics Showing the Top Five Binding Conformations. **(A)** δ-Carene with Erythromycin; **(B)** Amoxicillin; **(C)** Docking of α-Pinene with Erythromycin; **(D)** α-Pinene with Amoxicillin **(E)** Docking of α-Pinene with δ-Carene.

The docking results suggested the interaction energy values were analyzed to determine the strength of the interactions. Lower (more negative) values of interaction energy indicate stronger interactions. The docking structure for terpenoids- α-pinene, δ-carene and antibiotics- erythromycin, amoxicillin adopted, we have not included caryophyllene for the docking study (**Supplementary Figure 2**). The highest interaction was observed between δ-carene and erythromycin, with a total interaction energy value of -96.10 kcal/mol, suggesting a strong binding affinity between these two compounds (**Figure 5A**). This was followed by δ-carene with amoxicillin, which had an interaction energy value of -87.75 kcal/mol, indicating a significant interaction as well (**Figure 5B**). The α-pinene with erythromycin demonstrated an interaction energy of -83.58 kcal/mol, also reflecting a strong interaction but with amoxicillin had a slightly lower interaction energy value of -74.26 kcal/mol (**Figures 5C, D**). Lastly, the combination of α-pinene with δ-carene displayed an interaction energy of -57.02 kcal/mol, which, although lower compared to the other pairs (**Figure 5E**). These docking results suggest that δ-carene exhibits the highest potential for interaction with both erythromycin and amoxicillin, which could imply enhanced efficacy or synergistic effects in therapeutic applications involving these combinations.

## 4 Discussion

The study explores the antimicrobial potential of *C. oppositifolia* EOs and its terpenoid constituents, such as α-pinene, δ-carene, and caryophyllene, in combination with antibiotics like amoxicillin and erythromycin. The findings offer significant insights into their individual and synergistic effects against *S. aureus* and *E. coli*, contributing to our understanding of their potential applications in combating bacterial infections. The essential oil extracted from *C. oppositifolia* yielded 1.2% of the starting plant material, with a high purity of 95%. This extraction efficiency and purity are comparable to other studies on essential oils, which often report similar yields and purities, although minor losses due to volatility and oxidation were noted (Sharma et al., 2021). Despite these losses, the EO’s integrity and suitability for further analysis were maintained. The TLC analysis confirmed the presence of key terpenoids—α-pinene, δ-carene, and caryophyllene—in the EO. These findings are consistent with previous research identifying these terpenoids in *C. oppositifolia* and other plant species (Basholli-Salihu et al., 2017; Hanuš and Hod, 2020). The individual antimicrobial activity of these terpenoids demonstrated varying efficacy, with δ-carene showing the most potent effect against both *S. aureus* and *E. coli*. This is in line with other studies reporting δ-carene’s significant antimicrobial properties (Swamy et al., 2016; Guimarães et al., 2019). When combined with antibiotics, the terpenoids exhibited synergistic effects. For *S. aureus*, the combination of δ-carene and erythromycin resulted in the largest zone of inhibition, aligning with the findings of synergistic effects between terpenoids and antibiotics observed in similar studies (Mahfouz et al., 2023). Similarly, the combination of α-pinene with amoxicillin showed enhanced inhibition against *E. coli*, highlighting the potential for these combinations to improve therapeutic efficacy. The MIC values revealed that δ-carene had the lowest MICs for both bacterial strains, indicating its superior antimicrobial activity. These results are consistent with other studies that have reported δ-carene as a potent antimicrobial agent (Roman et al., 2023). Caryophyllene and α-pinene also demonstrated effective antimicrobial properties, although with higher MIC values compared to δ-carene (Valdivieso-Ugarte et al., 2019). The time-kill assay provided a detailed understanding of the bactericidal effects of the terpenoids and their combinations. All tested compounds, both individually and in combination with antibiotics, were effective in achieving complete bacterial kill at various time points. This is particularly noteworthy for the combination of α-pinene, δ-carene, and amoxicillin, which showed sustained bactericidal activity. Similar trends have been observed in other studies evaluating the time-kill kinetics of terpenoids and antibiotics (da Silva Rivas et al., 2012). The FTIR and UV spectroscopy analyses revealed significant changes in the functional groups and absorption spectra of the terpenoid-antibiotic combinations. The observed peak shifts and modifications suggest potential interactions or complex formations between these molecules. These findings are supported by other studies that have used FTIR and UV spectroscopy to investigate the interactions between terpenoids and antibiotics (Huang et al., 2022). The presence of new peaks or disappearance of existing ones in the spectra indicates possible changes in the molecular structures or electronic environments, which could contribute to the observed synergistic effects. Docking studies further elucidated the interaction energies between terpenoids and antibiotics. The strongest interactions were observed between δ-carene and erythromycin, with an interaction energy of -96.10 kcal/mol. This suggests a high binding affinity and potential for enhanced therapeutic efficacy. The results align with other docking studies that have explored similar interactions (Zlotnikov et al., 2023). Several limitations should be considered in interpreting these results. First, while the study provides valuable insights into the antimicrobial potential and interactions of the terpenoids and antibiotics, the *in- vitro* nature of the assays may not fully represent the *in-vivo* conditions. The effects observed in laboratory settings may differ from those in a physiological context due to factors such as metabolism and bioavailability. Study focused on a limited number of terpenoids and antibiotics. The inclusion of additional compounds could provide a more comprehensive understanding of potential synergistic effects and interactions. Study did not explore the mechanisms of action of the terpenoids and antibiotics in detail. Additional research, such as assessing bacterial cell wall integrity and intracellular targets, would provide deeper insights into how these compounds exert their antimicrobial effects. FTIR and UV spectroscopy analyses suggest possible interactions, further structural characterization and validation using techniques like NMR and X-ray crystallography could provide more definitive evidence of complex formation and interaction mechanisms.

## 5 Conclusion

The study demonstrates the significant antimicrobial potential of *C. oppositifolia* terpenoids and their synergistic effects with antibiotics. The results highlight the potential for developing novel combination therapies to enhance antimicrobial efficacy. However, further research is needed to validate these findings *in vivo*, explore additional terpenoid and antibiotic combinations, and elucidate the detailed mechanisms of action.

## Data Availability

The original contributions presented in the study are included in the article/**Supplementary Material**. Further inquiries can be directed to the corresponding authors.
