# Supplementary material for: Terpenoids as principal bioactive compound of Cissampelos oppositifolia essential oils: enhancing synergistic efficacy with conventional antibiotics
Source: Front Cell Infect Microbiol. 2024 Nov 28;14:1481656. doi: 10.3389/fcimb.2024.1481656 (PMC11634866; doi:10.3389/fcimb.2024.1481656)
Supplement: Supplementary file 1 [file DataSheet1.pdf]

Supplementary Table 1: Detailed Collection Data for 72 Plant Specimens, Including *C. oppositifolia*, from Two Locations in Himachal Pradesh, India

S. No.	Plant name	Parts used	Photo of the parts used
1	*Zanthoxyllum armatum* (Rutaceae)	leaves, seeds	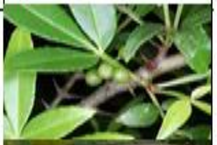
2	*Emblica officinalis* (Euphorbiaceae)	leaves	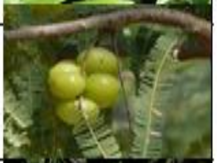
3	*Punica granatum* (Punicaceae)	Fower, leaves	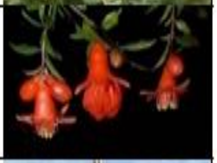
4	*Pinus roxberxyhii* (Pinaceae)	Bark, Laeves	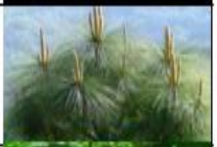
5	*Momordica charntia* (Cucurbitaceae)	laeves	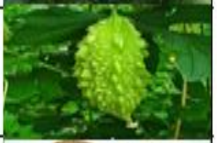
6	*Terminalia arjuna* (Combretaceae)	Bark	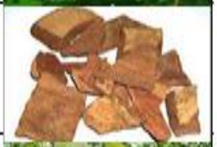
7	*Aegle marmelos* (Rutaceae)	Fruit	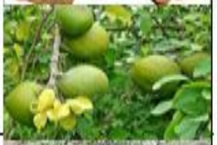
8	*Thymus vulgaris* (Lamiaceae)	Whole plant	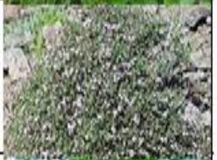
9	*Populous canadensis* (Salicaceae)	Leaves	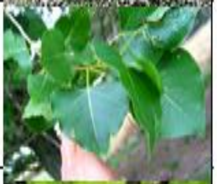
10	*Berberis aristata* (Berberidaceae)	Fruit, Leaves	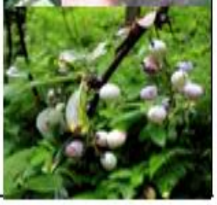

S. No.	Plant name	Parts used	Photo of the plant parts used
11.	*Cannabis sativa* (Cannabidaceae)	Leaves	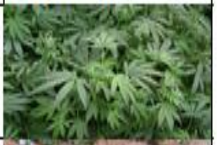
12	*Rhus cotinus* (Anacardiaceae)	Leaves	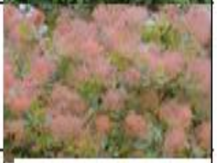
13	*Zingiber officinale* (Zingiberaceae)	Tuber	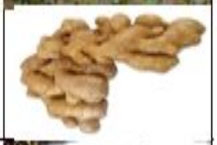
14	*Piper nigrum* (Piperaceae)	Fruit	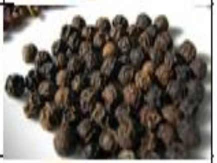
15	*Curcuma longa* (Zingiberaceae)	Tuber	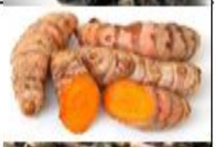
16	*Syzygium aromaticum* (Myristicaceae)	Flower bud	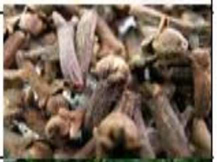
17	*Colebrookea oppositifolia* (Lamiaceae)	Leaves, Axial part, stem	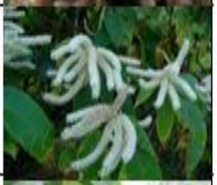
18	*Musa paradisiaca* (Musaceae)	Leaves	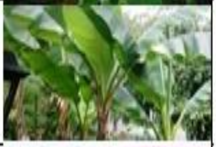
19	*Allium cepa* (Liliaceae)	Bulb	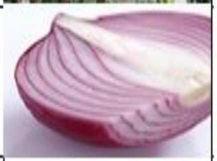
20	*Myristica fragrans* (Myristicaceae)	Flower, Fruit	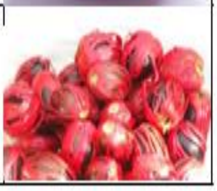

S. No.	Plant name	Parts used	Photo of plant used
21	*Euphorbia hirta* (Euphorbiaceae)	Whole plant	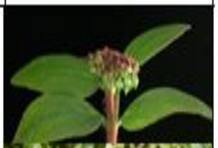
22	*Jaglans regia* (Juglandaceae)	Laeves	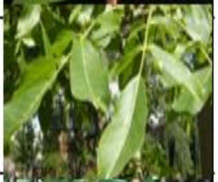
23	*Callistemon citrinus* (Myrtaceae)	Leaves	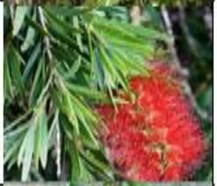
24	*Risinus communis* (Euphorbiaceae)	Leaves	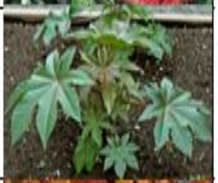
25	*Cinnamonum tamala* (Laureceae)	Bark	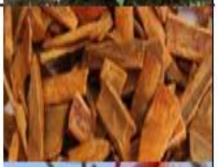
26	*Bombax ceiba* (Malvaceae)	Flower	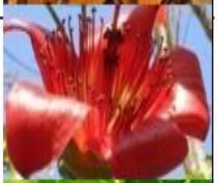
27	*Carissa spinarum* (Apocynaceae)	Leaves	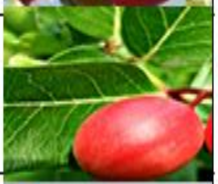
28	*Santalum album* (Santalaceae)	Bark	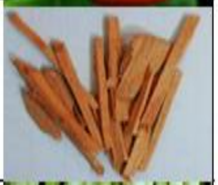
29	*Cymbopogon citrates* (Poaceae)	Oil	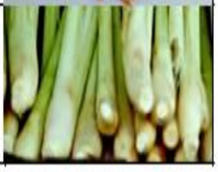
30	*Ruta gravelons* (Rutaceae)	Fruit	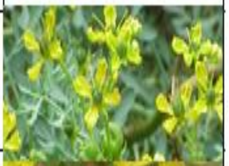
31	*Hypericum perforatum* (Hypericaceae)	Flower	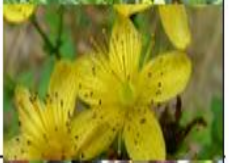
32	*Eugenia jambolana* (Myrtaceae)	Leaves	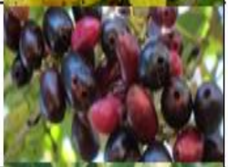
33	*Withania somnifera* (Solanaceae)	Fruit, Leaves	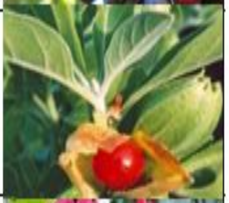
34	*Rhododendron arboreum* (Ericaceae)	Flower	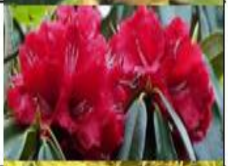
35	*Cuscuta reflexa* (Convolvulaceae)	Stem	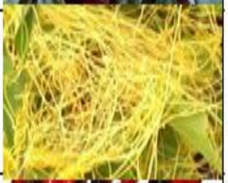
36	*Abrus precatorius* (Fabaceae)	Seeds	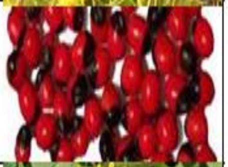
37	*Taraxacum officinale* (Compositae)	Leaves	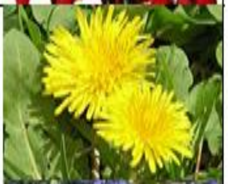
38	*Ajuga reptans* (Lamiaceae)	Flower	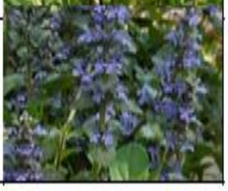

S. No.	Plant name	Parts used	Photo of the plant part used
39	*Ferula asafoetida* (Umbelliferae)	Latex	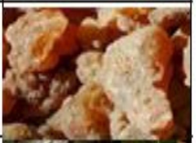
40	*Catharanthus roseus* (Apocynaceae)	leaves	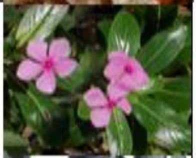
41	*Nigella sativa* (Ranunculaceae)	Seeds	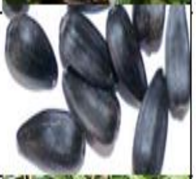
42	*Lawsonia alba* (Lythraceae)	Leaves	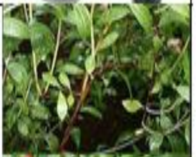
43	*Capsicum annum* (Solanaceae)	Fruit	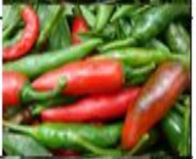
44	*Datura metel* (Solanaceae)	Fruit	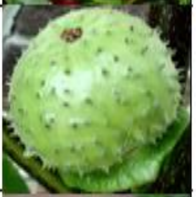
45	*Chenopodium album* (Chenopodiaceae)	Leaves	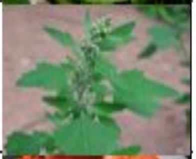
46	*Podophyllum hexandrum* (Berberidaceae)	Seeds	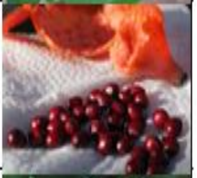
47	*Vitex negundo* (Lamiaceae)	Leaves	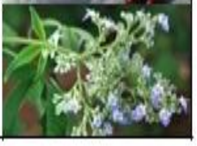
48	*Ageratum conyzoides* (Asteraceae)	Flower	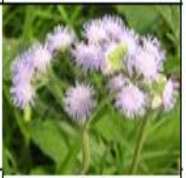
49	*Trachyspermum ammi* (Apiaceae)	Seeds	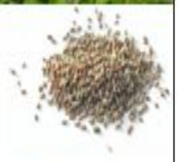
50	*Pelargonium hortorum* (Geraniaceae)	Leaves	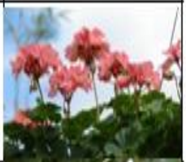
51	*Cinnamomum camphora* (Lauraceae)	Leaves	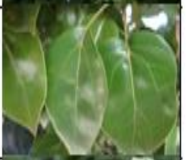
52	*Parthenium hysterophorus* (Asteraceae)	Whole plant	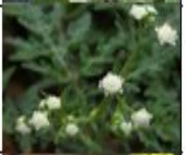
53	*Brassica oleracea* (Brassicaceae)	Flower	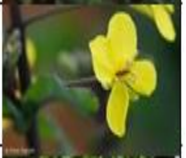
54	*Oenothera biennis* (Onagraceae)	Flower	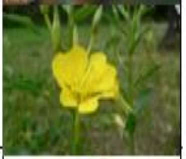
55	*Mimosa pudica* (Fabaceae)	Seeds	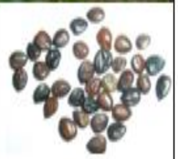
56	*Brassica nigra* (Cruciferease)	Seeds	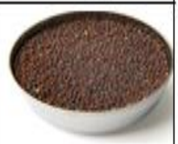

S. No.	Plant name	Parts used	Photo of the plant part used
57	*Colocasia esculenta* (Araceae)	Tuber	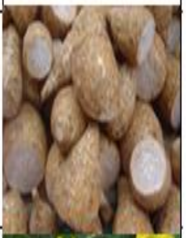
58	*Chrysanthemum indicum* (Asteraceae)	Flower	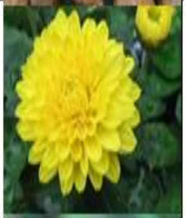
59	*Mentha viridis* (Lamiaceae)	Leaves	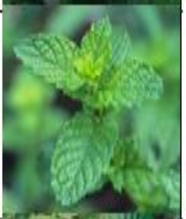
60	*Berginia ciliata* (Saxifraganceae)	Leaves	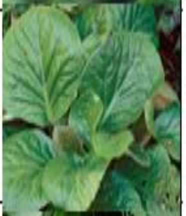
61	*Urtica dioica* (Urticaeae)	Leaves	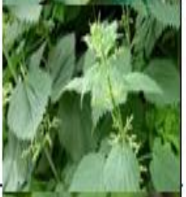
62	*Trigonellia foenum-graecum* (Fabaceae)	Leaves	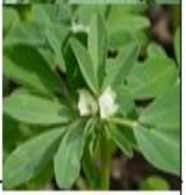
63	*Prunus amygdalus* (Rosaceae)	Oil	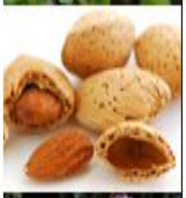
64	*Bauhinia variegata* (Fabaceae)	Flower	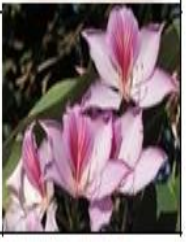

S. No.	Plant name	Parts used	Photo of the plant part used
65	*Camellia sinensis* (Theaceae)	Leaves	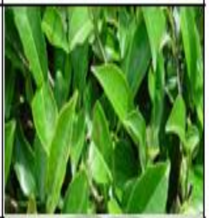
66	*Allium sativum* (Liliaceae)	Bulb	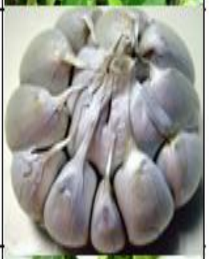
67	*Tinospora cordifolia* (Menispermaceae)	Leaves	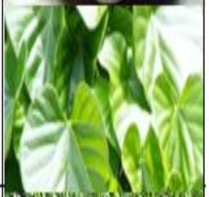
68	*Lavadula angustifolia* (Arecaceae)	Leaves	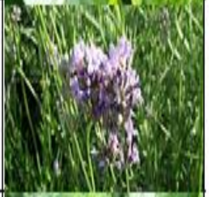
69	*Cichorium intybus* (Asteraceae)	Leaves	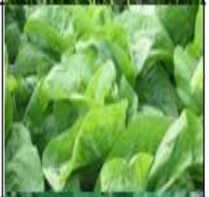
70	*Asclepias curassavica* (Asclepiadaceae)	Leaves, Flower, Stem	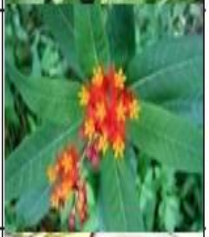
71	*Cocos nucifera* (Arecaceae)	Oil	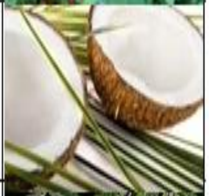
72	*Azardica indica* (Meliaceae)	leaves	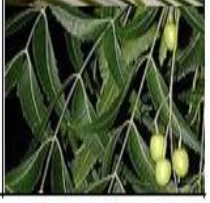

A.

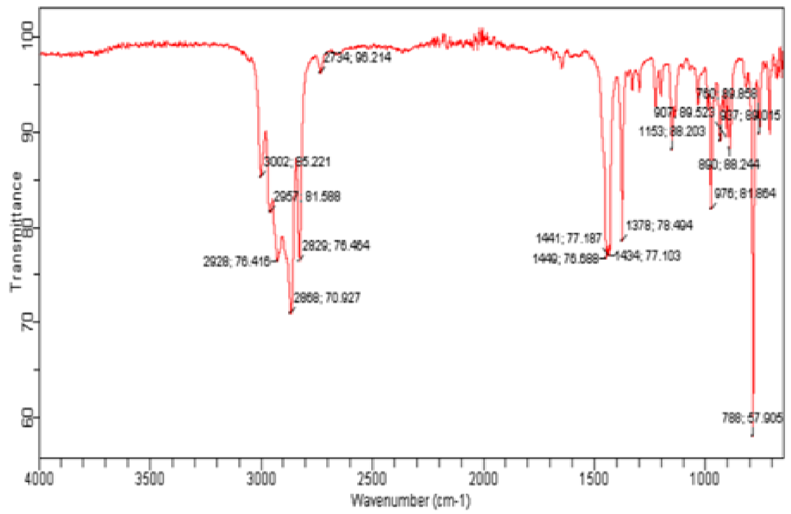

B.

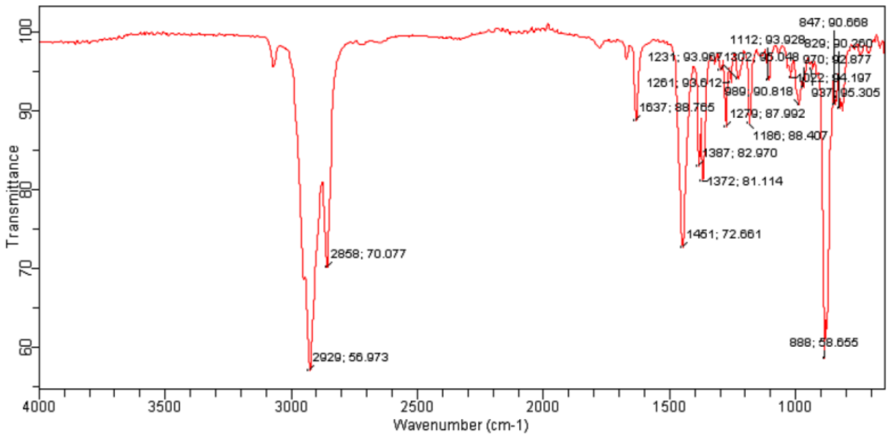

C.

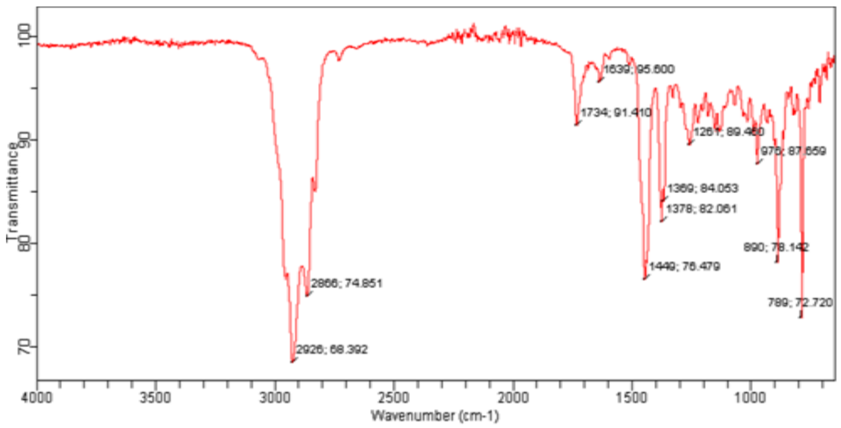

Supplementary Figure 1: FTIR Spectra Analysis of Teraponid A: carene; B: - pinene; C: caryophyllene

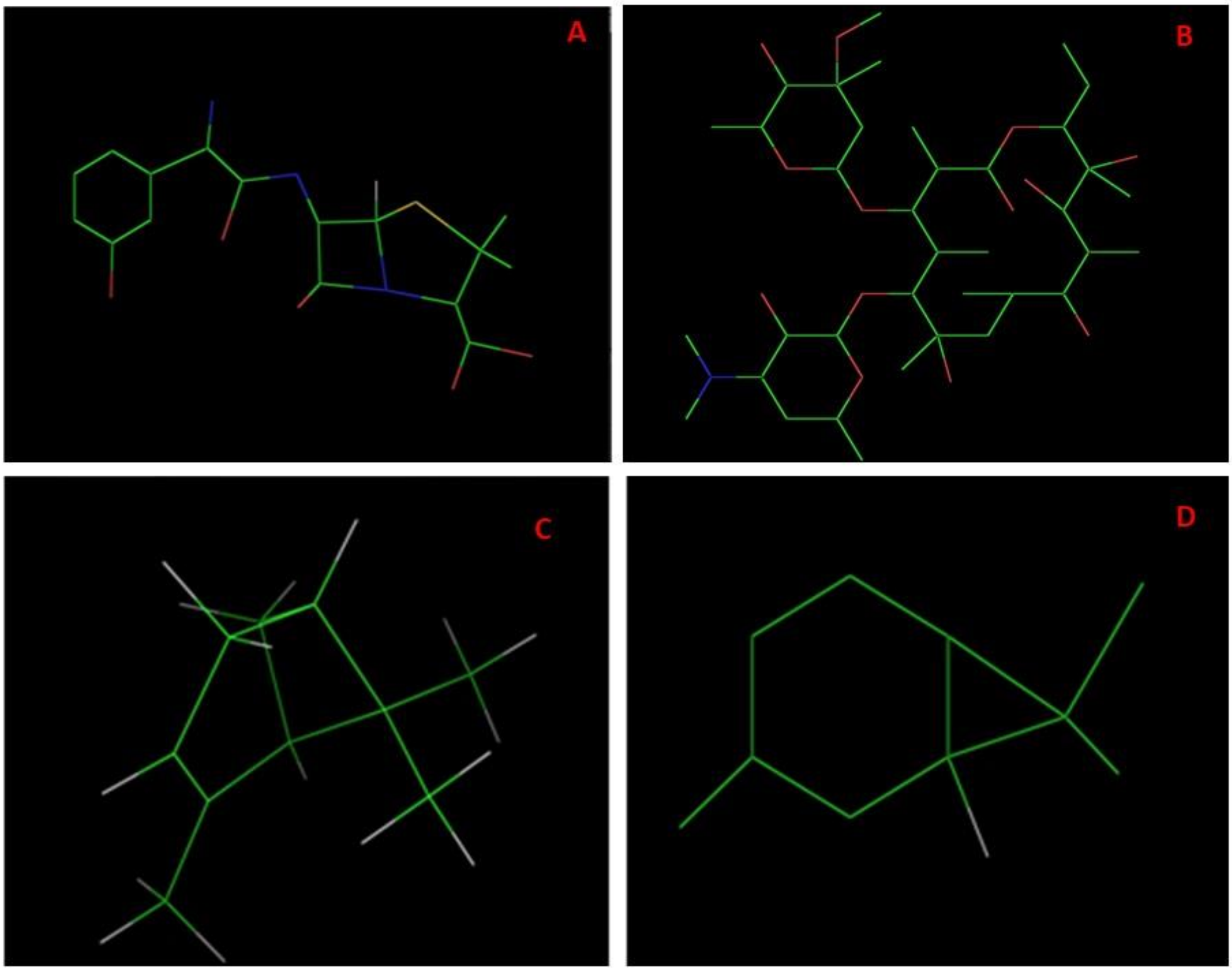

Supplementary Figure 3: The docking structure for terpenoids- A: α-pinene; B: δ-carene and antibiotics- C: erythromycin; D: amoxicillin
